# Current evidence, clinical applications, and future directions of transcranial magnetic stimulation as a treatment for ischemic stroke

**DOI:** 10.3389/fnins.2023.1177283

**Published:** 2023-07-18

**Authors:** Li Zhou, Yaju Jin, Danli Wu, Yongdan Cun, Chengcai Zhang, Yicheng Peng, Na Chen, Xichen Yang, Simei Zhang, Rong Ning, Peng Kuang, Zuhong Wang, Pengyue Zhang

**Affiliations:** ^1^Key Laboratory of Acupuncture and Massage for Treatment of Encephalopathy, College of Acupuncture, Tuina and Rehabilitation, Yunnan University of Traditional Chinese Medicine, Kunming, China; ^2^Kunming Municipal Hospital of Traditional Chinese Medicine, Kunming, Yunnan, China

**Keywords:** transcranial magnetic stimulation, ischemic stroke, underlying mechanisms, clinical applications, challenges, literature search and methods

## Abstract

Transcranial magnetic stimulation (TMS) is a non-invasive brain neurostimulation technique that can be used as one of the adjunctive treatment techniques for neurological recovery after stroke. Animal studies have shown that TMS treatment of rats with middle cerebral artery occlusion (MCAO) model reduced cerebral infarct volume and improved neurological dysfunction in model rats. In addition, clinical case reports have also shown that TMS treatment has positive neuroprotective effects in stroke patients, improving a variety of post-stroke neurological deficits such as motor function, swallowing, cognitive function, speech function, central post-stroke pain, spasticity, and other post-stroke sequelae. However, even though numerous studies have shown a neuroprotective effect of TMS in stroke patients, its possible neuroprotective mechanism is not clear. Therefore, in this review, we describe the potential mechanisms of TMS to improve neurological function in terms of neurogenesis, angiogenesis, anti-inflammation, antioxidant, and anti-apoptosis, and provide insight into the current clinical application of TMS in multiple neurological dysfunctions in stroke. Finally, some of the current challenges faced by TMS are summarized and some suggestions for its future research directions are made.

## Literature search and methods

**Figure 1:**
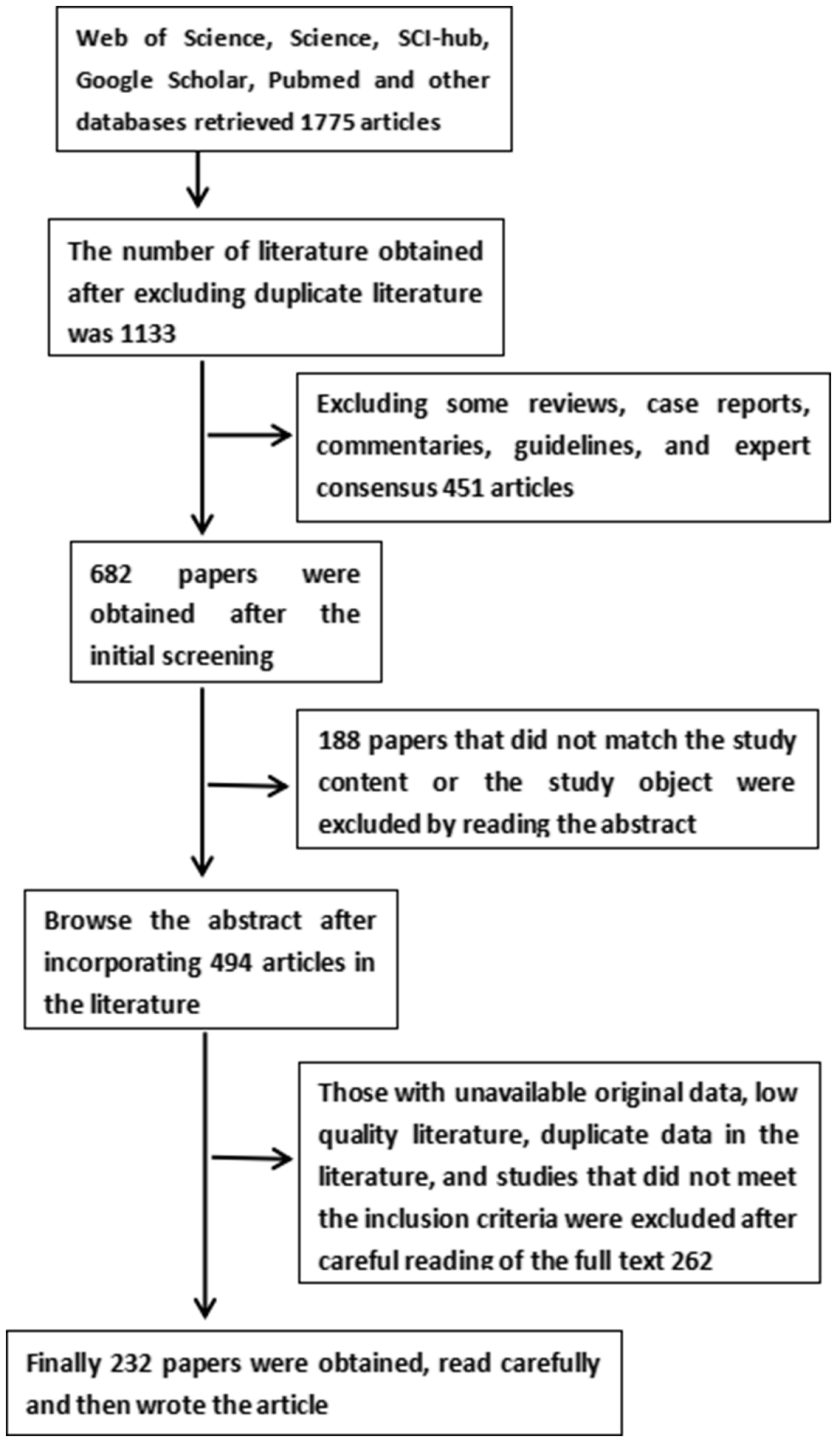
Flow chart of literature search.

We retrieved a large amount of literature from Web of Science, Science, SCI-hub, Google Scholar, Pubmed and other databases to search for keywords such as stroke, ischemic stroke, cerebrovascular accident, noninvasive brain stimulation technique (NIBS), transcranial magnetic stimulation (TMS), repetitive transcranial magnetic stimulation, single pulse transcranial magnetic stimulation (spTMS), paired pulsed transcranial magnetic stimulation (ppTMS), theta burst repetitive TMS (TBS), interhemispheric inhibition, cerebral ischemia–reperfusion injury (CIRI), neurotransmitters, excitatory neurotransmitters, glutamate, brain-derived neurotrophic factor (BDNF), Ca^2+^, neurogenesis, blood–brain barrier (BBB), astrocytes, microglia, oxidative stress injury, apoptosis, upper limb function, lower extremity function, speech, swallowing, cognition, post-stroke depression, spasticity, central post-stroke pain, adverse effects of TMS, and epilepsy, and kicked out studies that reported only protocols, trials that had not yet been completed, and caseloads of less than 8. A total of 136 randomized controlled trials, 23 experimental basic studies, and 5 meta-analyses were finally included (see Figure 1 for details).

## Introduction

Stroke is a cerebrovascular disease of the middle-aged and elderly due to impaired cerebral blood flow circulation leading to tissue damage. With the economic and social development of countries, the occurrence of stroke is becoming younger and younger. Stroke includes two types of stroke: ischemic and hemorrhagic stroke, ischemic stroke accounts for 87% of all strokes and is characterized by high morbidity, disability and mortality, and is the leading cause of disability and death induced by human diseases [NCD Risk Factor Collaboration (NCD-RisC), 2021]. According to stroke epidemiology in 2020, the number of stroke patients worldwide is expected to increase by about 30 million per year, which will place a heavy burden on the global society and economy (The Lancet Neurology, 2021). After ischemic stroke, the key treatment strategy to save damaged brain tissue is to achieve cerebral blood flow recanalization. Commonly used treatments include intravenous thrombolysis (Higashida et al., 2003), endovascular therapy (Chen C. J. et al., 2015) and bridging therapy (Albuquerque, 2014), all of which are reperfusion therapies based on thrombolysis and thrombus retrieval as a clinical tool to mitigate further neurological deterioration in stroke patients by salvaging the semidark zone tissue. However, due to the strict time window for cerebral blood flow reperfusion therapy, only about 15% of stroke patients are able to achieve cerebral blood flow recanalization (Wahlgren et al., 2016). Although the time window for thrombolytic therapy in stroke patients has been extended to 24 h in recent years (Ragoschke-Schumm and Walter, 2018), reperfusion therapy still faces great challenges and risks and may even further aggravate neurological deficits in stroke patients, such as neurological disorders in cognition (Delavaran et al., 2017), speech (Schumacher et al., 2019), swallowing (Sreedharan et al., 2022), and sensorimotor function (D'Imperio et al., 2021). Therefore, with the trend of increasing stroke incidence year by year, it is extremely necessary to propose a new adjuvant therapy to improve neurological dysfunction and enhance activities of daily living in stroke patients.

TMS is a non-invasive neurostimulation technique that is painless, non-invasive and needs to be used while the patient is awake, and consists of two main components: the main unit and the treatment cap or probe (with coils). During treatment, the probe is placed on the cerebral cortex to be stimulated and an electric current is passed through the coil to generate a magnetic field, followed by an induced current that affects the cerebral cortex, thus achieving a transient enhancement or suppression of cortical neuroexcitability (Kobayashi and Pascual-Leone, 2003; Rossini and Rossi, 2007; Kesikburun, 2022). TMS has been reported to have neuroprotective effects in a variety of neurological disorders, such as Alzheimer’s disease (Mimura et al., 2021), Parkinson’s (Chen and Chen, 2019), multiple sclerosis (Aloizou et al., 2021), depression (Toth et al., 2022), vascular dementia (Nardone et al., 2011), and stroke (Tosun et al., 2017). Some studies have shown that TMS combined with a variety of rehabilitation treatments can significantly reduce neurological dysfunction, improve the ability to perform activities of daily living, and improve the quality of life of stroke patients (Dionísio et al., 2021; Hoonhorst et al., 2021; Zong et al., 2022). This suggests that TMS is a feasible adjunctive treatment for stroke.

There are differences in the site and timing of TMS stimulation for different dysfunctions in stroke. Currently, the commonly used stimulation sites in stroke are primary motor cortex (M1), left dorsolateral prefrontal cortex (DLPFC), superior temporal gyrus, inferior frontal gyrus, and secondary somatosensory cortex (S2) (Lee et al., 2022). In addition, there are brain areas that are used as stimulation sites according to their functional counterparts. Among these stimulation sites, the primary motor cortex is by far the most commonly used stimulation site, presumably because M1 is the central regulatory point for motor control and functional decision making in the body (Lee et al., 2022); second, M1 is a multi-overlapping, multifunctional cortical area involved in multiple functions such as motor, cognitive, speech, and swallowing. Therefore, for stroke, a multifunctional disorder neurological disease, M1 is one of the common stimulation targets irreplaceable for TMS treatment of stroke disease. In addition, the duration of TMS stimulation time varies, and the determination of its treatment time is related to the acute and chronic stage of the disease, its severity, the patient’s tolerance level, and the treatment goal. However, although TMS has been widely used to improve neurological dysfunction after stroke, its potential mechanisms, optimal stimulation modality, site, frequency, and duration to promote neural repair are not clear and need to be further explored. Therefore, this review will summarize the potential mechanisms of TMS to improve post-stroke neurological function, review the relevant clinical applications of TMS in stroke patients, and summarize the current risks and challenges that remain with TMS.

### Classification of transcranial magnetic stimulation

TMS is not only a therapeutic tool for many neurological disorders, but also a common screening or diagnostic tool. When it is applied to the motor cortex of the brain, the target cortical muscles produce corresponding motor evoked potentials (MEP), and the MEP can be recorded in real time with surface electromyography (EMG) to determine the continuity and integrity of motor neural pathways. In addition, the EEG values recorded in combination with EEG are real-time readings of TMS-induced changes in cortical excitability. Thus, TMS can be used as a test tool to record changes in cortical neurological function and brain dynamics. Currently, TMS is often divided into three forms: single-pulse TMS (spTMS) (Henriette et al., 2022), paired-pulse TMS (ppTMS) (Du et al., 2014) and repetitive TMS (rTMS) (Klomjai et al., 2015).

The spTMS is a single-pulse stimulus that occurs every few seconds, and is commonly used to study individual motor thresholds as well as cortical neural excitability (Klomjai et al., 2015; Jarczok et al., 2021). Similarly, ppTMS is the exploration of intracortical circuit excitability (Boulogne et al., 2016) by generating two consecutive pulses of stimulation every few seconds, including both intracortical inhibition (ICI) and intracortical facilitation (ICF) endings, which ultimately lead to different courses of ICI and ICF depending on the intensity of the stimulus before and after the two pulses, and the interval between the two stimuli (Hanajima et al., 2002; Ilić et al., 2002; Mcclintock et al., 2011). The rTMS is a repetitive pulse stimulation of the same frequency and intensity for a certain period of time, and after the cumulative sum of these repetitive pulse stimuli reaches the threshold of evoked neural activity, the stimulated site can briefly produce neural excitatory or inhibitory activity (Vlachos et al., 2012). If rTMS stimulation is repeated several times, then the effects produced can last longer and even form synapses that persist (Kricheldorff et al., 2022).

In addition, rTMS has been extended by simulating the firing pattern of hippocampal neurons with a new type of TMS, in which three pulses at 50 Hz occur at 200 ms intervals, called theta burst repetitive TMS (TBS) (Ferrarelli and Phillips, 2021). Compared with conventional rTMS, TBS can induce longer duration and more intense neural activity with low-intensity, short duration stimulation (Kricheldorff et al., 2022). TBS includes interstitial theta pulse stimulation (iTBS) and continuous theta pulse stimulation (cTBS) (Bai Z. et al., 2022). In general, iTBS increases neuronal excitability and enhances LTP-like effects, while cTBS induces LTD-like effects and suppresses neuronal excitability (Huang et al., 2009). However, not all iTBS and cTBS follow this pattern, as Xue et al. found that cTBS treatment upregulated the release of neurotrophic factors in stroke patients, increased the number of new neurons in the penumbra region, and promoted post-stroke neurogenesis (Zong et al., 2022). The reason for this heterogeneity may be due to the different neuronal populations finally activated by cTBS and iTBS (Volz et al., 2015), as well as the different recruitment of indirect (I) wave in the late corticospinal overhead jerk after stimulation (Di Lazzaro et al., 2008). In addition to TBS, rTMS includes a quadruple pulse stimulation (QPS) with four monophasic pulse stimulation repeated every 5 s (Nakatani-Enomoto et al., 2012), which produces a bidirectional effect when acting on the motor cortex of the brain, i.e., short interval QPS enhances neuroplasticity and long interval QPS inhibits neuroplasticity (Hamada et al., 2008; Ni and Chen, 2008). Although QPS is similar to ppTMS, Masashi et al. found that QPS was more effective than ppTMS in inducing motor cortical neuroplasticity with better persistence and specificity than ppTMS (Hamada et al., 2007).

### Effects of transcranial magnetic stimulation parameters on cortical excitability

Impaired excitability of the motor cortex in the hemisphere of the post-stroke lesion results in limited movement of the hemiplegic side of the limb (Liepert et al., 2000), one of the causes of this impairment is dysregulation of interhemispheric inhibition after stroke (Xu et al., 2019). In a healthy state, both hemispheres regulate each other’s cortical excitability via the corpus callosum pathway, so that the bilateral interhemispheric excitability is maintained in balance and no over inhibition occurs (Duque et al., 2005). In contrast, after stroke, interhemispheric inhibition is imbalanced, and cortical excitability in the lesioned hemisphere is reduced by oversuppression of excitability, while cortical excitability in the non-lesioned hemisphere is increased. This leads to a variety of neurological sequelae after stroke and reduces the quality of patient survival (Bütefisch et al., 2008; Wahl et al., 2016; Patel et al., 2019). Therefore, regulation of interhemispheric excitability balance is an indispensable remedy to improve post-stroke neurological dysfunction (Lin et al., 2020; Sharma et al., 2020).

The world’s first transcranial magnetic device was developed in 1985 by Barker et al. (1985) as a tool to study changes in excitability of the motor cortex of the brain after stimulation. So how is the target cortical excitability modulated when TMS is used to treat stroke patients? In general, high frequency TMS (HF-TMS) in the lesioned hemisphere increases cortical excitability and low frequency TMS (LF-TMS) in the non-lesioned hemisphere decreases cortical excitability (Iyer et al., 2003; Kim et al., 2004; Chen M. et al., 2015; Nordmann et al., 2015). However, is there a boundary between HF-TMS and LF-TMS? Most studies consider low frequency ≤ 1 Hz and high frequency > 1 Hz (Modugno et al., 2003; Filipović et al., 2010; Fried et al., 2017; Tugin et al., 2021); some studies consider high frequency ≥ 5–20 Hz (Somaa et al., 2022); and a few other researchers consider high frequency ≥ 3 Hz (Kubis, 2016; Tian and Izumi, 2022). Thus, it seems that LF-TMS is relatively stable compared to HF-TMS, which are in the range of 0-1 Hz, and the majority of studies mostly use 1 Hz (Heide et al., 2006; Meng et al., 2020). And some studies have shown that the inhibitory effect of 1 Hz-rTMS on cortical excitability is the most pronounced (Muller et al., 2014). However, LF-rTMS at 0.25 Hz (Muller et al., 2014), 0.1 Hz (Chen et al., 1997), 0.3 Hz (Cincotta et al., 2003), 0.6 Hz (Khedr et al., 2004), and 0.9 Hz have also been used in some studies (Kricheldorff et al., 2022).

The modulation of target cortical excitability by TMS is also influenced by stimulus parameters (Lang et al., 2006; Taylor and Loo, 2007; Jung et al., 2008; De Jesus et al., 2014). Within a certain range, cortical excitability produces stronger and more sustained effects with increasing TMS pulse number (Tang et al., 2019). In other words, the number of pulses is proportional to cortical excitability within a certain limit (Touge et al., 2001; Peinemann et al., 2004), which also varies from individual to individual. Therefore, when patients receive TMS for the first time, they should be pre-stimulated to explore the optimal TMS parameters. In addition to this, cortical excitability is influenced by stimulation intensity and time. The differences caused by stimulus intensity are mainly related to interindividual motor thresholds (MT) (D'amico et al., 2020; Rizzo et al., 2020). Cortical excitability tends to change at the MT intensity node, with stimulus intensities greater than MT increasing MEP amplitude and thus target cortical excitability; conversely, target cortical excitability usually decreases when stimulus intensities are less than MT (Modugno et al., 2001; Fitzgerald et al., 2002). However, who is more dominant in the effect of TMS frequency and intensity on cortical excitability? Or are they of equal status? Heide et al. (2006) found that rTMS at a frequency of 1 Hz and an intensity of 115% of resting motor threshold (RMT) applied to primary motor cortex (M1) for several minutes resulted in reduced cortical excitability (Meng et al., 2020). This suggests that frequency may be more important for the modulation of cortical excitability. However, a study by Gabrielle et al. using HF-rTMS at three low intensities (70, 80 and 90% of active motor threshold (AMT)) found that only stimulus intensities up to 90% AMT promoted target cortical excitability, whereas both 70 and 80% AMT inhibited cortical excitability (Todd et al., 2006). This suggests that it is the modulation of cortical excitability by stimulus intensity that may be more important. In summary, we suggest that frequency and intensity may be equally important for the modulation of cortical excitability, but that the expected effects differ due to inter-individual differences in MT. Of course, these are our speculations and the exact mechanisms are not yet clear. In addition, target cortical excitability is also influenced by the stimulus waveform (Arai et al., 2005), with monophasic pulses having a stronger cortical excitatory effect due to preferential activation of neuronal populations in the same direction compared to biphasic pulses (Arai et al., 2007).

### Potential mechanisms of transcranial magnetic stimulation for stroke

TMS is known to promote the improvement of different neurological functions through different stimulation sites. For example, sites such as M1 (Bai G. et al., 2022), dorsolateral prefrontal cortex (DLPFC; Hara et al., 2021), and parietal cortex (Rushworth and Taylor, 2006) can improve motor, cognitive (Zhang et al., 2022), and speech dysfunctions in stroke patients. However, there is no more comprehensive summary of how TMS facilitates the improvement of neurological function after stroke. Therefore, in the following, we will briefly describe the potential mechanisms of TMS from these aspects.

#### Transcranial magnetic stimulation regulates the concentration of excitatory neurotransmitter-glutamate

Neuroexcitotoxicity after ischemic stroke is a key link leading to neuronal death, which can induce a series of pathological cascade reactions that eventually lead neurons to apoptosis or necrosis. Neuroexcitotoxicity is mainly caused by Ca^2+^ overload after cerebral ischemia and hypoxia, massive release of excitatory neurotransmitter glutamate and excessive activation of ionotropic glutamate receptors. Therefore, glutamate receptor inhibitors have been used clinically to block the over-activation of glutamate receptors and thus reduce the damage caused by neuroexcitotoxicity after stroke, but the effect is not very obvious. In recent years, it has been found that TMS can regulate human excitatory neurotransmitter-glutamate levels, which may be one of the potential targets to prevent or mitigate post-stroke excitotoxicity.

TMS can modulate neural activity by regulating glutamate concentrations in the nervous system, e.g., 10 Hz-rTMS significantly downregulates glutamate levels in the striatum (dorsal and NAc; Poh et al., 2019). Similarly, Eugenia et al. demonstrated that rTMS reduces the concentration of neurotransmitters such as glutamate, striatal serine, threonine, sarcosine, and aspartate in the nervous system (Poh et al., 2019). It has also been shown that 0.5-Hz-rTMS leads to significantly higher levels of glutamate in the hippocampus and striatum, but glutamate levels in the hypothalamus are reduced. These results suggest that the regulation of glutamate concentration by rTMS may be influenced by the stimulation site, frequency, and the concentration of glutamate levels in various brain regions, and that its main purpose is to regulate glutamate concentration to a normal range; thus, the mechanism by which rTMS regulates glutamate concentration varies in different brain regions. However, this is our speculation and more studies are needed to verify it (Yue et al., 2009).

In addition, TMS can promote glutamate uptake by neurons and decrease glutamate accumulation between neurons by upregulating glutamate receptor activity or expression. For example, Adeline et al. observed that 1 Hz-rTMS promotes upregulation of glutamate receptor 5 (GluA5) receptor expression (Etiévant et al., 2015). Similar studies have shown that TMS promotes the upregulation of the number and density of α-amino-3-hydroxy-5-methyl-4-isoxazolepropionic acid (AMPA) receptors of glutamate receptor 1 (GluA1; Vlachos et al., 2012; Lenz et al., 2015). Furthermore, in addition to regulating glutamate receptors, glutamate transporter proteins can also regulate extracellular glutamate levels. Therefore, it has been suggested that TMS regulation of glutamate concentration may also be related to the expression of glutamate transporter proteins. A study showed that rTMS promotes upregulation of the expression of genes of glutamate transporter proteins such as EAAT4, GLAST, GLT1, and EAAC24 (Ikeda et al., 2019). This is the first study in which rTMS was observed to regulate the expression of glutamate transporter proteins, providing a potential direction for future studies on the mechanism of rTMS.

#### Transcranial magnetic stimulation promotes neurogenesis

Neurogenesis plays a pivotal role in promoting improved neurological function after stroke. Brain-derived neurotrophic factor (BDNF), a member of the neurotrophic factor family, is one of the most important regulators of activity-dependent neuroplasticity (Luo et al., 2017). In healthy states, BDNF is involved in promoting neurogenesis in the central nervous system in addition to nutritional support of nerve cells (Tan et al., 2021). For example, it promotes the formation of dendritic spines (Chaturvedi et al., 2020) and facilitates synaptic long-term potentiation (LTP) (Leal et al., 2014). In addition, BDNF regulates the balance of excitatory and inhibitory neurotransmitters in the brain (Karantali et al., 2021). In addition, it has been shown that patients with significantly lower serum BDNF levels and increased brain infarct volume after stroke have a worse functional outcome of stroke (Qiao et al., 2017; Duan et al., 2018). It is thus clear that regulation of BDNF expression has an irreplaceable role in promoting post-stroke neurogenesis.

In recent years, TMS has become an integral part of stroke rehabilitation treatment strategies. Ma et al. (2014) found that 110% RMT, 1 Hz-rTMS activated the BDNF/tyrosine kinase receptor B (TrkB) pathway and promoted neuroplasticity; however, as the stimulation intensity increased to 150% RMT, the BDNF/TrkB pathway was inhibited, the number of synapses decreased, the density and thickness thinned, and neurogenesis was suppressed. In addition, Guo et al. (2014) found that TMS significantly upregulated miR-25 expression and promoted the proliferation of neural stem cells (NSCs) in the subventricular zone of stroke patients. It has also been shown that the proliferation and differentiation of newborn neural stem cells to the ischemic lesion area after stroke is regulated by BDNF, and overexpression of BDNF accelerates the recruitment of newborn neural stem cells and their migration to the ischemic brain tissue area (Young et al., 2011; Jansson et al., 2012). In addition to the above possible mechanisms, it has also been shown that iTBS regulates intrasynaptic Ca^2+^ concentration by affecting N-methyl-D-aspartate (NMDA) activity, thereby increasing intrasynaptic transmission efficiency and promoting LTP-like effects (Saudargiene et al., 2015; Diao et al., 2022). It is thus clear that TMS promotes improved neurological function after stroke and is closely related to multiple pathway-mediated neurogenesis.

#### Transcranial magnetic stimulation promotes vascular regeneration

Vascular injury and blood–brain barrier (BBB) disruption caused by ischemia after stroke induces brain edema formation, which exacerbates post-stroke neurological dysfunction (Fagan et al., 2005; Rodríguez-Yáñez et al., 2006). Therefore, promoting angiogenesis becomes one of the potential targets to rescue ischemic brain tissue and improve neurological function. Astrocytes are the most abundant neuronal cells in the brain (Yu G. et al., 2021) and have two phenotypes: classical activation (A1) and alternative activation (A2) (Liddelow et al., 2017). In general, both types of astrocytes play important physiological roles both in healthy and injured conditions. A1-type astrocytes promote the release of inflammatory factors to induce inflammatory responses, while A2-type astrocytes increase the release of angiogenesis-related factors such as transforming growth factor-β (TGFβ) and vascular endothelial growth factor (VEGF) to promote angiogenesis (Blackburn et al., 2009; Liddelow and Barres, 2017).Mark et al. found that chronic intracerebroventricular infusion of VEGF significantly promoted vascular collateral circulation formation and increased vascular density (Harrigan et al., 2002). Sun et al. (2003) also found that increasing VEGF levels stimulated vascular regeneration after cerebral ischemia. In summary, promoting upregulation of VEGF levels by promoting the polarization of A1-type astrocytes to A2-type astrocytes and thus promoting vascular regeneration may be one of the molecular mechanisms to improve neural repair after stroke.

In recent years, with the rapid development of TMS, studies on the mechanism of TMS to improve neurological function after stroke have been increasing. One study showed that 10 Hz-rTMS promoted the polarization of A1-type astrocytes to A2-type, thereby reducing infarct volume and promoting neurological recovery in MCAO rats (Hong et al., 2020). Similarly, Zong X. et al. (2020a) reported that rTMS promoted the polarization of A2-type astrocytes, elevated the levels of angiogenesis-related factors TGFβ, VEGF, and hypoxia-inducible factor 1α (HIF-1α), and increased the density and volume of neovascularization in the penumbra region of MCAO rats. The above studies suggest that one of the mechanisms of neuroprotective effects of TMS is the induction of A2-type astrocyte polarization and promotion of vascular regeneration.

#### Anti-inflammatory properties of transcranial magnetic stimulation

The immune inflammatory response is rapidly activated after stroke and continues throughout the stroke period. In general, the early inflammatory response contributes to neuroprotection by phagocytosis of dead cell debris or certain harmful toxic substances (Wen et al., 2006), but prolonged inflammatory factor infiltration is detrimental to post-stroke neuronal survival (Qin et al., 2019). Therefore, reducing the inflammatory response and preventing excessive release of inflammatory factors are essential to improve the internal environment for neuronal survival after stroke.

Microglia act as neurological macrophages in the brain and play an important role in maintaining the homeostasis of the intracerebral environment (Ma et al., 2017). There are two phenotypes, classical activated (M1) and alternative activated (M2), which play different roles in response to different stimuli during different pathological stages of ischemic stroke (Hu et al., 2015). M1 microglia release tumor necrosis factor (TNF-α), interleukin 1β (IL-1β), interferon γ (IFN-γ), interleukin 6 (IL-6), inducible nitric oxide synthase (iNOS), matrix metalloproteinase 9 (MMP9), matrix metalloproteinase 3 (MMP3), and other pro-inflammatory mediators, induce BBB permeabilization and accelerate ischemic neuronal death (Kreutzberg, 1996; Yenari et al., 2010). In contrast, M2 microglia release anti-inflammatory and pro-angiogenic mediators such as interleukin 10 (IL-10), TGF-β, and VEGF, which exert neuroprotective effects (Ponomarev et al., 2013; Prinz et al., 2014). Once stroke occurs, M1 and M2 microglia are activated to release both pro-inflammatory and anti-inflammatory mediators, and the resistance of these two mediators determines the fate course of neuronal cells in the ischemic infarct zone (Hu et al., 2012; Zhao et al., 2017). Therefore, regulation of M1/M2 microglia polarization during different stages of stroke may be one of the mechanisms regulating the brain microenvironment and promoting neural repair.

TMS is currently used as a common intervention for stroke rehabilitation. Zhao et al. (2019) found that rTMS significantly reduced IL-1β and TNF-α serum levels in patients. In the MCAO model, the 10 Hz-rTMS experimental group activated more M2 microglia and reduced activation of M1 microglia than the no sham stimulation group (Hong et al., 2022). Immediately after, this study also showed that rTMS significantly increased microglia let-7b-5p levels and inhibited their downstream NF-kB signaling pathway, thereby reducing the size of cerebral infarcts in MCAO rats; in addition, *in vitro* experiments showed that administration of rTMS to microglia increased the concentration of interleukin 10 (IL-10) and decreased the concentration of TNF-α in the culture medium, while knockdown of let-7b -5p reversed these phenomena (Hong et al., 2022). This study repeatedly verified from *in vivo* and *ex vivo* experiments that rTMS ameliorates neurological dysfunction in MCAO model mice by promoting M2-type microglia polarization, regulating the let-7b-5p/NF-kB signaling pathway, and attenuating the inflammatory response. Similarly, it was shown (Luo et al., 2022) that iTBS significantly reduced IL-1β, interleukin 17A (IL-17A), TNF-α, and IFN-γ concentrations in MCAO mice by inhibiting M1 activation, promoting M2 polarization, and downregulating Toll-like receptor 4 (TLR4)/NF-κB/NLR family Pyrin domain-containing protein 3 (NLRP3) signaling pathway. Elevated IL-10 concentrations reduced neuromotor dysfunction in MCAO mice, whereas removal of microglia after using the inhibitor eliminated the efficacy of iTBS application, which inversely verified that iTBS ameliorated post-stroke neurological dysfunction by regulating the balance of M1/M2 phenotype of microglia.

#### Antioxidant properties of transcranial magnetic stimulation

Achieving blood flow recanalization as soon as possible after stroke is a top priority to salvage the ischemic semidark zone. However, cerebral ischemia–reperfusion is prone to cerebral ischemia–reperfusion injury (CIRI) because it has a strict time window (Zhao et al., 2022). Therefore, it is important to explore the underlying mechanisms of CIRI to explore potential targets for stroke therapy. It has been reported that CIRI injury is mainly caused by the reactivation of mitochondrial aerobic respiration after blood flow recanalization, which generates large amounts of reactive oxygen species (ROS) and induces oxidative stress in neuronal cells (Orellana-Urzúa et al., 2020; Yu S. et al., 2021). Therefore, reducing ROS release after stroke and enhancing the antioxidant capacity of the body are key steps to reduce CIRI injury. Studies have shown that TMS promotes functional repair in a variety of neurological diseases in association with its powerful antioxidant effects (Post et al., 1999; Medina-Fernandez et al., 2017). For example, Hui et al. found that rTMS attenuates CIRI injury by activating the nuclear factor E2-related factor 2 (Nrf2) signaling pathway, upregulating the expression of antioxidant proteins such as Nrf2, heme oxygenase 1 (HO-1), and superoxide dismutase 1 (SOD1), and reducing oxidative stress (Liang et al., 2021).

#### Anti-apoptotic properties of transcranial magnetic stimulation

The tissue blood supply around the ischemic infarct foci (penumbra zone) in the early post-stroke period is still maintained at 20–40% while retaining some oxygenated metabolic activity (Hou et al., 2019). Therefore, salvaging the penumbra for a certain period of time is essential to reduce the volume of post-stroke cerebral infarction. However, it has been found that within hours or days after cerebral ischemia, the ischemic penumbra in response to ischemic–hypoxic stress induces some neuronal cells toward apoptosis and releases toxic substances that further accelerate neuronal death (Vexler et al., 1997; Radak et al., 2017). Thus, apoptosis, which is activated in the acute phase of stroke, is a major obstacle to the remodeling of neuronal cell structure and function in the ischemic penumbra region after stroke.

A recent study showed that the potential mechanism by which rTMS promotes neurological recovery in MCAO rats is related to the inhibition of premature apoptosis of neuronal cells in the penumbra region (Gao et al., 2010; Yoon et al., 2011). Yamei et al. showed that rTMS upregulated B lymphocytoma-2 gene (Bcl-2) levels and decreased Bcl-2-related X protein (Bax) expression significantly inhibited apoptosis, thereby improving neurological recovery in MCAO rats (Guo et al., 2017). Similarly, a similar study also found that TMS combined with electroacupuncture treatment inhibited post-ischemic neuronal apoptosis by significantly reducing cystein-3 (Caspase-3) levels and increasing Bcl-2 mRNA expression levels (Li et al., 2012). In addition, rTMS can reduce mitochondrial damage, maintain mitochondrial membrane integrity, inhibit the activation of the mitochondria-dependent apoptotic cystatase-9 (Caspase-9)/Caspase-3 signaling pathway, and reduce neuronal death (Sasso et al., 2016; Zong et al., 2020b).

### Clinical application of transcranial magnetic stimulation in stroke diseases

Stroke is one of the leading causes of neurological disability in the world (Saini et al., 2021). Once stroke occurs, a variety of neurological complications follow, leading to severe limitations in patients’ activities of daily living (Chohan et al., 2019). Therefore, exploring the application of new technologies in stroke rehabilitation is pivotal to provide more clinical experience in the future treatment of stroke patients; in addition, it will provide a better understanding of the advantages and disadvantages, indications, contraindications, and precautions of this technology and promote better service of this technology to society. In recent years, more and more studies have reported that TMS plays an irreplaceable role in the neurological rehabilitation process of stroke, involving upper and lower limb motor function, speech, swallowing, cognitive function, post-stroke depression, spasticity, and central post-stroke pain (see Table 1 for details). However, the potential mechanisms by which TMS improves the above neurological dysfunctions? and whether the stimulation frequency, intensity, duration and site are consistent are unclear? Therefore, the clinical application of TMS in stroke rehabilitation is briefly described below, and each of these queries will be explored.

**Table 1:** Clinical application of transcranial magnetic stimulation in post-stroke sequelae.

Type of Research	Stimulation site	Stimulation frequency	Stimulation time	Sample size	Amount of effect	Results	References
Clinical Studies	Lesioned hemisphere	10 Hz	1 time/day, 5 days/week, 4 weeks in total	9	9	Improvement in upper limb motor function	Ni et al. (2022)
		5 Hz	1 time/day, 6 days/week, 10 times in total	20	18		Hosomi et al. (2016)
		10 Hz	1 time/day, 20 min/time, 13 days in total	19	19	Improvement in lower limb walking speed	Kakuda et al. (2013)
		5 Hz	1 time/day, 15 min/time, 3 times/week, total 3 weeks	6	6		Wang et al. (2019)
		10 Hz	1 time/day, 40 min/day for 2 weeks	15	15	Motor function, muscle strength improvement	Wang et al. (2020)
		10 Hz	1 time/day, 20 min/time, 5 times/week, 4 weeks in total	18	16	Improved cognitive function	Yin et al. (2020)
		20 Hz	1 time/day, 20 min/time, 5 times/week for 2 weeks	10	10		Cha et al. (2022)
		50 Hz (iTBS)	1 time/day, 20 min/time, 5 times/week, total 6 weeks	22	21		Chu et al. (2022)
		10 Hz	1 time/day, 30 min/time, 5 times/week for 2 weeks	11	11	Improvement in swallowing function	Park et al. (2017)
		5 Hz	1 time/day, 30 min/day for 4 weeks	15	14		
			1 time/day, 10 min/time, for 2 weeks	9	9		Park et al. (2013)
			1 time/day, 30 min/time, 5 times/week for 2 weeks	30	23		Liu et al. (2022)
		3 Hz	1 time/day, 20 min/time, 5 times/week for 2 weeks	32	21		Jiao et al. (2022)
			1 time/day, 10 min/day, 5 days in total	14	14		Khedr et al. (2009)
		10 Hz	1 time/day, 10 min/time, 10 times in total	10	10	Improvement in speech function at 2-month follow-up	Hu et al. (2018)
	Undiseased hemisphere	1 Hz	1 time/day, 20 min/time, for 2 weeks	11	11	Improvement in upper limb motor function	Noh et al. (2019)
			1 time/day, 10 min/time, 5 times/week for 2 weeks	23	21		Pan et al. (2019)
			1 time/day, 5 days/week, total 8 weeks	20	15		Qin et al. (2023)
			1 time/day, 3 days/week, 10 times in total	10	10		Barros Galvão et al. (2014)
			1 time/day, 5 days/week for 2 weeks	17	14		Gottlieb et al. (2021)
			1 time/day for 15 days	21	21		Long et al. (2018)
			1 time/day, 20 min/day for 2 weeks	9	9	Improvement of fine motor function of the hand	Tosun et al. (2017)
			1 time/day, 20 min/day, 5 days/week, total 2 weeks	20	20		Aşkın et al. (2017)
			1 time/day, 20 min/time, 10 times in total	9	9		Tretriluxana et al. (2013)
			1 time/day, 30 min/time, 6 times/week for 2 weeks	20	20		Yang et al. (2022)
			5 times/day for 1 week	10	7	Lower extremity motor function and spasticity improvement	Rastgoo et al. (2016)
			1 time/day, 6 days/week, 4 weeks in total	30	30		Li et al. (2021)
			1 time/day, 30 min/time, 10 times in total	14	12		Wang et al. (2012)
			1 time/day, 40 min/day for 2 weeks	15	15	Motor function, muscle strength improvement	Wang et al. (2020)
			1 time/day, 5 days/week, total 8 weeks	18	18	Improved cognitive function	Yingli et al. (2022)
			1 time/day, 5 days/week, 4 weeks in total	30	28	Language function improvement	Bai G. et al. (2022)
			1 time/day, 30 min/time, 5 times/week for 2 weeks	6	6		Haghighi et al. (2017)
			1 time/day, 10 min/day, 10 times in total	10	10		Hu et al. (2018)
			1 time/day, 20 min/time, for 15 days	36	31		Ren et al. (2019)
			1 time/day, 40 min/time, 6 times/week, 10 times in total	24	24		Abo et al. (2012)
			1 time/day, 30 min/time, 5 times/week, 15 times in total	13	8		Waldowski et al. (2012)
	Bilateral hemispheres	10 Hz and 1 Hz	1 time/day, 5 days/week, 4 weeks in total	9	9	Improvement in upper limb motor function	Ni et al. (2022)
			1 time/day, 40 min/day for 2 weeks	15	15	Motor function, muscle strength improvement	Wang et al. (2020)
		5 Hz and 1 Hz	1 time/day, 30 min/day for 4 weeks	15	15	Improvement in swallowing function	

### Motor dysfunction

#### Upper extremity

Motor dysfunction of the upper extremity after stroke is mainly characterized by reduced movement, limb coordination and dexterity and accounts for 55–75% of all stroke patients (Wolf et al., 2006). Compared with the lower extremity, the recovery of motor function in the upper extremity after stroke progresses relatively slowly and is mainly concentrated in the first 6 months. Due to the weak awareness of rehabilitation in most patients, by the time they start to intervene in rehabilitation, they have already missed the golden time of upper limb function recovery. Therefore, it is crucial to raise the patients’ awareness of rehabilitation and to find an effective complementary therapy.

Recently, several randomized controlled trials have shown that rTMS modulates cortical excitability and restores interhemispheric inhibitory balance to improve motor dysfunction after stroke (Kim et al., 2006; Long et al., 2018). A study by Avenanti et al. showed that HF-rTMS significantly increased cortical excitability in the lesioned hemisphere, thus promoting improved distal upper limb movements, finger dexterity and coordination in stroke patients (Kim et al., 2006). However, during stimulation of upper limb motor function, one study found that bilateral TMS (high frequency on the lesioned side and low frequency on the unlesioned side) was more beneficial than unilateral TMS in improving upper limb motor function (Long et al., 2018). However, another meta-analysis found that this view was only supported in the acute phase of stroke, whereas in the subacute and chronic phases, TMS on the lesioned and unlesioned side alone achieved better outcomes (Chen et al., 2022). This suggests that promoting cortical excitability in the lesioned hemisphere during the acute and subacute phases of stroke is the main strategy to improve neurological function in the upper limbs of patients, whereas inhibiting cortical excitability in the unlesioned hemisphere and reducing its inhibition of the lesioned hemisphere during the chronic phase is the main mechanism to improve neurological function. In conclusion, the different periods of the disease are also important factors in determining the TMS treatment plan.

In addition, a similar picture exists for different stimulation modalities, e.g., TBS is more beneficial for the recovery of upper limb motor function in the acute phase of stroke, whereas rTMS is more effective in the subacute and chronic phases (Chen et al., 2022). In addition, it is worth noting that rTMS promotes the recovery of fine motor function of the upper limbs in stroke patients by indirectly modulating the excitability of the corticospinal tract in addition to directly modulating the excitability of the cerebral cortex, however, this process is heavily dependent on the integrity of the corticospinal tract (Wu et al., 2023). Therefore, when applying rTMS to improve upper limb motor function after stroke, the integrity of the corticospinal tract can be tested before developing an appropriate rTMS protocol. This process can be referred to the results of the study by Birute et al. For example, low-frequency rTMS is more adapted for stroke patients with high corticospinal tract (CST) integrity; in contrast, high-frequency rTMS showed more significant clinical effects in patients with low CST integrity (Wang et al., 2022).

#### Lower extremity

It is well known that both lower extremities play an important role in human motor function, as demonstrated by walking and balance coordination (Paul et al., 2007). After stroke, patients often suffer from lower extremity motor dysfunction, gait abnormalities, and other sequelae that severely reduce patients’ family and social participation. TMS is an emerging therapy for stroke treatment that can increase walking speed (Li et al., 2018; Vaz et al., 2019), correct gait symmetry (Wang et al., 2019), reduce lower extremity muscle spasticity (Naghdi et al., 2015; Rastgoo et al., 2016), and increase balance and motor control (Wang et al., 2012; Kang et al., 2020) to improve lower limb function in stroke patients.

A randomized controlled trial showed that rTMS significantly improved walking, motor control, and motor function in stroke patients, a finding further emphasized by late follow-up data (Veldema and Gharabaghi, 2022). This study followed the theory of interhemispheric inhibition, using HF-rTMS to promote reactivation of cortical excitability in the diseased hemisphere and LF-rTMS to reduce cortical excitability in the unlesioned hemisphere and alleviate its overinhibition of the diseased hemisphere, thereby readjusting the bilateral interhemispheric inhibitory balance. In this study, the bilateral rTMS regimen was superior to the unilateral rTMS regimen, i.e., bilateral rTMS > HF-rTMS on the lesioned side > LF-rTMS on the unlesioned side (Veldema and Gharabaghi, 2022). In fact, in most studies, it has been demonstrated that bilateral rTMS regimens are superior to unilateral regimens, which is similar to the fact that in some diseases combined treatment is superior to monotherapy, which is uncontroversial. Then, leaving aside the bilateral rTMS regimen, why HF-rTMS on the lesioned side > LF-rTMS on the unlesioned side? We believe that after brain injury, reduced excitability in the lesioned hemisphere is the main cause of interhemispheric inhibition imbalance, and therefore, reactivation of cortical excitability in the lesioned hemisphere may be the main strategy to restore interhemispheric inhibition balance compared to reducing excitability in the unlesioned hemisphere. However, this dominance is not invariable, and depending on the etiology, location of the lesion, the urgency of the disease, and individual patient differences, some patients show that reducing excitability in the unlesioned hemisphere is more likely to promote interhemispheric inhibitory homeostasis. As found in a meta-analysis, LF-rTMS in the unlesioned hemisphere produced more clinically significant effects than HF-rTMS in the lesioned hemisphere during the chronic phase of stroke (Chen et al., 2022).

In addition, there is some controversy about TMS improving lower limb function in stroke, for example, a meta-analysis showed that both high and low frequency rTMS had a positive effect on the gait speed of stroke patients (Vaz et al., 2019). However, this was not supported by a study by Raffaella et al. who found that 20 Hz HF-rTMS, while significantly improving lower limb motor function in chronic stroke patients, did not increase the patients’ walking speed (Chieffo et al., 2014). In addition, Ying et al. found that LF- rTMS (1 Hz) administered in the unlesioned hemisphere did not significantly improve motor and walking function in stroke patients (Huang et al., 2018). However, in another study the opposite result was obtained, that LF-rTMS in the unlesioned hemisphere improved muscle spasticity in patients’ lower extremities, thus promoting improved motor function in the lower extremities (Rastgoo et al., 2016), this idea was also supported by the studies of Soofia et al. and Liu et al.(Naghdi et al., 2015; Liu et al., 2021). And this research variability may arise from the fact that walking is a complex process consisting of several parameters such as walking speed and angle, step width, stride width, and step length. And the regulation of these parameters is modulated by multiple neural systems such as cortical, subcortical and spinal integrated networks. As when walking, multiple sites such as primary sensorimotor areas, primary motor areas, supplementary motor areas, basal ganglia and cerebellar earthworms were detected to be activated with increased cerebral blood flow (Veldema and Gharabaghi, 2022). This suggests that TMS improves lower extremity motor function in patients not only by the TMS protocol, but also by the different stimulation sites and the interactions between brain regions. Therefore, future studies are needed to create more and more beneficial evidence for the various differences arising from TMS application and further advance TMS.

### Cognitive impairment

One study reported that one third of stroke patients have varying degrees of cognitive impairment, referred to as post-stroke cognitive impairment (PSCI), and PSCI can rapidly develop into dementia in a short period of time, which will seriously reduce the quality of life of patients (Li Y. et al., 2020). Therefore, active rehabilitation measures to prevent PSCI from developing into dementia are extremely important to improve the quality of life and social participation of stroke patients.

Studies have shown, TMS may improve cognitive function in patients with PSCI through anti-inflammation and increased cerebral blood flow (Cha et al., 2022). For example, Takatoshi et al. showed that 10 Hz-rTMS promotes improved memory, attention and executive function in PSCI patients by increasing blood perfusion in ischemic brain regions (Hara et al., 2017). The dorsolateral prefrontal cortex (DLPFC) is often used as a stimulation site for TMS to improve PSCI and plays an important role in modulating higher cognitive functions such as memory, attention, and executive functions (Bressler and Menon, 2010). Yin et al. (2020) showed that left DLPFC HF-rTMS significantly improved executive function in PSCI patients. Similarly, according to the theory of interhemispheric inhibitory balance, right-sided DLPFC LF-rTMS also promotes improved cognitive and memory functions in patients with PSCI (Lu et al., 2015; Yingli et al., 2022). Furthermore, a meta-analysis showed that rTMS improved PSCI better when stimulated for longer than 4 weeks and at a stimulation intensity in the range of 80–110% MT (Yin et al., 2020; Wang et al., 2022). However, in addition to rTMS, iTBS is one of the commonly used stimulation modalities to improve PSCI, and left-sided DLPFC iTBS significantly promotes improvements in cognitive functions such as executive function and semantic comprehension in patients with PSCI compared to sham stimulation (Chu et al., 2022; Li et al., 2022).

### Swallowing disorders

More than 65% of new stroke patients each year have dysphagia (Martino et al., 2005). Although dysphagia is a self-resolving complication in most cases, 11–50% of patients will have permanent dysphagia without intervention (Kumar et al., 2010). Therefore, it is necessary to prevent dysphagia from becoming a permanent sequel with external means.

Studies have shown that TMS treatment applied in the M1 brain region can promote the improvement of swallowing function after stroke (Du et al., 2016). However, M1 area covers multiple neurological functions, and how to precisely locate the functional area of M1 swallowing becomes a major obstacle. Until Li S. et al. (2020) used functional MRI (fMRI)-guided TMS in the motor cortex of the brain to excite surface electromyography of the labial orbicularis muscle and detected motor evoked potentials (MEPs) in the submandibular complex (SMC) muscle as a way to locate MSC targets in the M1 swallowing functional area. This study provides a more precise target stimulation site for TMS to improve post-stroke swallowing dysfunction. However, as the process is an individualized one, it may target different individuals with slightly different MSC target locations. Therefore, repositioning of SMC targets for different individuals is necessary for effective improvement of post-stroke swallowing function.

Guided by fMRI, TMS can more accurately and effectively promote improvement in swallowing function, especially HF-TMS (Du et al., 2016; Liao et al., 2017). Xiang et al. showed that HF-rTMS improved dysphagia in stroke for a longer duration and with more significant effects than LF-rTMS (Liao et al., 2017). It is also noteworthy that LF-rTMS (1 Hz) only improved appetite in stroke patients compared to conventional swallowing function treatment, while it did not seem to have a significant effect on the recovery of swallowing function (Ünlüer et al., 2019). Furthermore, Cheng et al. (2015) showed that swallowing function was significantly improved in chronic stroke patients after 3,000 magnetic pulses of 5 Hz-rTMS applied to the tongue motor area of the diseased hemisphere for 2 weeks of continuous treatment. However, in another study, similar results were not obtained with the same stimulation parameters, and no improvement in swallowing function was observed in stroke patients at 2, 6, or even 12 months follow-up after the end of treatment (Cheng et al., 2017). This is in contrast to the findings of Cheng et al. (2015). These two refuting studies suggest that the clinical efficacy of TMS may vary even within the same disease depending on the severity of the disease, individual differences between patients, or other external factors, and therefore, exploring the optimal clinical efficacy of TMS still requires a long time to figure out.

Similarly, a randomized controlled trial observed that the recovery of swallowing function was significantly better in the bilateral stimulation group (simultaneous application of rTMS treatment to the lesioned and unlesioned side of the hyoid muscle) compared to the unilateral stimulation group (500 10 Hz-rTMS pulses applied to the lesioned side of the hyoid muscle for 2 weeks; Park et al., 2017). This finding was supported by a study by Momosaki et al. (2014). However, in another meta-analysis, no significant difference in swallowing function was found between subgroups with different stimulation sites (lesioned hemisphere, unlesioned hemisphere, or bilateral hemisphere) (*p* = 0. 53; Wen et al., 2022). We believe that this phenomenon may be due to partial case error and that perhaps the meta-analysis is more convincing compared to the control trial and could continue to be illustrated by continuing to increase the sample size.

### Aphasia

Post-stroke aphasia (PSA) is a common acquired language disorder in patients with acute or subacute stroke, which can lead to varying degrees of impairment in four areas of listening, reading, and writing (Hara and Abo, 2021). In the long term, this will lead to loss of self-confidence in life and may induce post-stroke depression in severe cases (Hilari et al., 2010). Therefore, early treatment of patients with PSA is a key step in preventing post-stroke depression.

TMS has been used since 2005 for the treatment of aphasia in patients with chronic stroke (Gholami et al., 2022). Compared with conventional speech therapy, TMS significantly promotes improvements in naming, comprehension, repetition, and writing in PSA patients (Yao et al., 2020). In general, TMS treatment protocols vary among PSA patients with different language dysfunctions. For example, LF-rTMS is more beneficial for the improvement of function in spontaneous speech and auditory comprehension in PSA patients (Hu et al., 2018). In addition, the site of stimulation is a major factor in TMS parameters that affects clinical efficacy. The superior temporal gyrus is the main lesioned brain region in sensory aphasia (also known as Wernicke’s aphasia), where severe impairment in spoken language comprehension is the main clinical manifestation; therefore, the superior temporal gyrus is preferentially chosen as the stimulation site when the patient is Wernicke’s aphasic (Ren et al., 2019). Similarly, another typical aphasia, motor aphasia (also known as Broca’s aphasia), has a lesion in the inferior frontal gyrus, and when this area is stimulated, the patient’s spontaneous speech and repetition components are significantly improved (Ren et al., 2019). This shows that it is perfectly feasible to select the site of TMS stimulation according to the lesioned brain areas of different aphasia types. However, although the lesioned brain area can be used as a stimulation site for PSA patients, it is not clear whether this site is the best stimulation site for TMS, and there are no relevant studies to prove this idea. Therefore, future studies can try to compare different stimulation sites for the same aphasia type to observe the treatment effect of both groups and verify whether the lesioned brain area is the best stimulation site for PSA patients.

### Post-stroke depression

Post-stroke depression (PSD) is the most common neuropsychiatric sequelae in stroke patients, with approximately one-third of new patients experiencing PSD each year (Shen et al., 2017). Currently, antidepressants are the most common treatment for PSD, but the clinical time to effect of pharmacological treatment is long and only some patients improve significantly after pharmacological treatment. Therefore, the search for a treatment other than medication is promising for promoting improvement in PSD. And as early as 2008, it was demonstrated that rTMS treatment in the dorsolateral prefrontal cortex to promote improvement in PSD is safe and effective with few side effects (Duan et al., 2023). Therefore, rTMS is undoubtedly a safe and reliable alternative for those patients with PSD for whom psychotherapy is ineffective or for whom pharmacological treatment has serious side effects.

The traditional rTMS regimen of 5 days of treatment per week for 4–6 weeks has been reported to have significant positive effects in patients with chronic major depression (Duan et al., 2023). However, the duration of this rTMS regimen is relatively long, and is it possible to achieve the same effect while reducing the duration of treatment in a clinical setting for certain patients with poor compliance or who cannot adhere to daily rTMS sessions? Jessica et al. found that accelerated rTMS (20 Hz, 110% RMT, 5 sessions per day for 4 days) significantly reduced PSD and sustained a positive effect at 3-month follow-up (Frey et al., 2020). In addition, this study showed that accelerated rTMS is also safe and feasible in patients with subacute PSD, with no risk of inducing epilepsy yet (Frey et al., 2020). However, because the study had only six cases, the number was small and a larger sample size is needed for validation. Based on this study, we can tentatively conclude that accelerated rTMS is indeed more effective than conventional rTMS in the acute subacute phase of PSD; this may be because in the early phase of stroke recovery, the body recovers neurologically faster, and therefore, seizing the opportunity for high-intensity intervention during that time is also a safe and feasible treatment option. This is similar to the fact that the first 6 months after stroke is a critical period for patients to recover upper limb function, and if that time window is not seized, the possibility of upper limb function recovery may be missed; because for most stroke patients, upper limb function is slow to recover, and if rehabilitation is not done well in the first 6 months, it is possible that later rehabilitation will have minimal effect on their neurological function improvement. However, is there a critical period for recovery of different neurological functions or are they all consistent? This is a key point that should be addressed. If we are clear about the critical period for each type of neurological recovery, we may be able to promote the recovery of neurological functions more effectively, even without unnecessary sequelae due to time problems.

### Spasticity

The early intervention of rehabilitative exercise training is the cornerstone of the future social reintegration of stroke patients. However, post-stroke spasticity (PSS), a complication that can seriously affect the recovery process, is a major disorder that severely affects motor training and is characterized by a speed-dependent increase in reflex tone. In general, not all stroke patients experience hypertonia as a clinical manifestation. About 30–80% of stroke patients are experiencing or are about to suffer from spasticity, which can cause pain, contracture, deformity, or even stiffness and immobility in the joints if not taken promptly (Wei et al., 2022).

Although numerous clinical studies have demonstrated that TMS significantly reduces post-stroke limb spasticity, a small number of studies have not yielded similar results, and they suggest that the reasons for this heterogeneity may be multifaceted, such as stimulation parameters, stimulation site, type of coil, and the severity and acute and chronic phase of stroke patients can affect the effect of TMS in reducing muscle spasticity in patients (Xia et al., 2022). Different types of coils have different degrees of influence in improving the spasticity state, with the figure-of-eight coil featuring higher focal stimulation having a significant effect in reducing spasticity symptoms compared to the H-type coil (Li et al., 2021). Second, the therapeutic effect of spasticity also varies depending on the different stimulation sites of TMS. In addition to the cerebral motor cortex, the cerebellum plays an important role in motor control and regulation of muscle tone stabilization. It was found that cerebellar cTBS can reduce muscle spasticity in stroke patients by regulating corticospinal excitability through the cerebellar-dentate thalamus-cortex pathway (Li et al., 2021). Therefore, cerebellar cTBS may be one of the more promising stimulation modalities among the methods to reduce muscle spasticity in the future development of TMS to reduce spasticity. However, it has been found that lesions in a common functional region connected to the bilateral nucleus accumbens and pallidum on the side of the lesion may be a potentially critical region contributing to PSS (Qin et al., 2022), so could this region be used as a TMS target to directly improve PSS? As we know, this region is located in the striatal area under the cortical overlay, which is a higher subcortical center that regulates a variety of complex neural functions such as motor, sensory and memory. As TMS is a non-invasive neurostimulation technique, if we want TMS to target the common area of bilateral nucleus accumbens and pallidum, we can only indirectly stimulate to this area through the cerebral cortex, so how can we exclude the influence of the cerebral cortex and the interaction between these two areas? It is also possible that this is one of the reasons why this region has not been used as a TMS target.

### Central post-stroke pain

Central post-stroke pain (CPSP) is one of the common neuropathic pain sequelae after cerebral ischemic injury, often manifesting as sensory hypersensitivity or sensory abnormalities at the site corresponding to the vascular lesion, which is easily confused with stroke-induced shoulder subluxation pain and muscle spasticity pain, resulting in patients who may miss the best early symptomatic treatment period and seriously reduce their quality of life. Currently, CPSP is mainly treated with pharmacological agents, such as anticonvulsants, which have good effects on improving the symptoms of CPSP (Bo et al., 2022), but when it comes to intractable CPSP, the drugs may only play the role of placebo and do not provide a good solution to the patient’s CPSP troubles. A recent study found that rTMS in the cerebral motor cortex had a significant effect on relieving recalcitrant CPSP (Malfitano et al., 2021), but the duration varied, which may be related to the site of ischemia, the period of disease progression, the site of stimulation, and individual patient differences. Therefore, there is a need to explore the therapeutic parameters related to the improvement of CPSP by TMS.

In addition, most studies suggest that the possible cause of CPSP is central de-inhibition or central imbalance due to ischemia (Ri, 2022), then restoration of abnormal cortical excitability by TMS may be one of the potential mechanisms for the relief of recalcitrant CPSP. As found in the study, 10 Hz HF-rTMS had significant analgesic effects in CPSP patients in all periods, and M1 HF-rTMS had better analgesic effects than M1 LF-rTMS (Leung et al., 2020; Malfitano et al., 2021). This suggests that in patients with CPSP, increasing cortical excitability in the lesioned hemisphere is far more effective than inhibiting cortical excitability in the unlesioned hemisphere, after all, the imbalance in the regulation of downstream somatosensory pathways due to reduced cortical excitability in the lesioned hemisphere is the most direct cause of CPSP. Therefore, restoration of cortical excitability in the lesioned hemisphere of CPSP patients is one of the potential mechanisms for the analgesic effect of rTMS. In addition, one study found that rTMS significantly relieved CPSP was associated with changes in serum BDNF levels, and an increase in BDNF levels reduced pain in CPSP patients (Zhao et al., 2021). In addition, rTMS increases the functional link between somatosensory pathways, which in turn has an analgesic effect (Kadono et al., 2021). The above studies suggest that the analgesic effect of rTMS in CPSP patients may be related to the modulation of neuroplasticity in the lesioned hemisphere and cortical excitability during the recovery period.

### Challenges and prospects

Although TMS is a commonly used stroke treatment today, there are some safety risks associated with the action of TMS in humans. For example, in a meta-analysis trial, 13 patients out of 273 subjects experienced adverse events such as headache, dizziness, rhinorrhea, syncope, and seizures (Qiao et al., 2022). Among them, epilepsy is the most serious sequelae of TMS and is currently the most controversial point for scientists to question TMS.

The TMS safety guidelines suggest that the occurrence of epilepsy is influenced by a variety of external and internal factors (Walton et al., 2021). External factors generally include errors present in TMS equipment, errors in the operation of medical personnel, and the adjustment of TMS-related parameters. In general, TMS devices need to be checked for their hardware devices when they are first used to prevent the occurrence of adverse reactions such as epilepsy due to inaccuracies in the devices (Bae et al., 2007). Secondly, the occurrence of adverse reactions is also related to TMS type, frequency, intensity, time, and coil shape. TMS protocols with high frequencies, intensities >120% MT, and short pulse intervals are more likely to induce epilepsy (Tendler et al., 2018; Rossi et al., 2021). However, what is the range of high frequencies? A study showed that TMS at 15 Hz, 120% MT, and 0.75 s pulse interval induced epilepsy (Tendler et al., 2018). Does that suggest that HF-TMS at 15 Hz or greater than 15 Hz necessarily leads to epileptogenesis? The answer does not seem to be the case, as a subset of clinical studies with TMS frequencies above 15 Hz or even up to 20 Hz did not induce epilepsy or other adverse effects (Chieffo et al., 2014). This suggests that TMS is not always accompanied by the occurrence of adverse reactions and that inter-individual differences between patients are a factor in its incidence. Second, coil type also affects the incidence of epilepsy, with the incidence of digital-8 coils inducing epilepsy at 3/1000 or less than 1%(Oberman et al., 2011). The incidence of digital-8 coils inducing epilepsy has also been reported to be in the range of 0.08/1000, while H-type coils affect epilepsy in the range of 0.12–0.43/1000 (Stultz et al., 2020). In this comparison, it seems that the figure-of-8 coil is a little safer, but TMS adverse effects are influenced by more factors, and we are not sure whether the interference of other factors has been discharged when the figure-of-8 and H coils were compared. Therefore, the conclusion still lacks some credibility. In addition, internal factors mainly refer to the variability of TMS for different individuals, including aspects such as history of disease and history of taking medications. Patients with a history of brain injury, epilepsy, and those who have taken antidepressants (Kreuzer et al., 2013) or antiepileptic drugs (Dobek et al., 2015) have been reported to be prone to epilepsy.

TMS-induced epilepsy is influenced by numerous factors; therefore, when a patient is first treated with TMS, the patient should be pre-evaluated for acceptability of TMS treatment and potential triggers for adverse reactions before developing a safe and reliable individualized TMS protocol for the patient. This includes the optimal parameters for TMS, the most suitable stimulation site, and precautions to be taken during the treatment. However, it is worth noting that the optimal parameters are not necessarily within the safe range, and in some patients the optimal parameters exceed the safe range of TMS without inducing an adverse event. Therefore, is it necessary to choose the optimal TMS parameters in such cases in a big but, or to choose the safe parameters of TMS for insurance purposes remains a clinical problem? In addition, some patients experience adverse reactions within the safe range. In summary, the triggering factors of TMS adverse reactions may also be influenced by other factors that have not yet been identified; therefore, further exploration of the triggering factors of TMS adverse reactions and prevention of side effects such as seizures in patients is still a direction of urgent research. In addition, although there are many animal experiments on TMS, few trials have been conducted to validate it in humans, which is undoubtedly a major obstacle to the development process of TMS.

## Conclusion

To date, there is a large body of clinical evidence supporting the value of TMS in improving post-stroke neurological deficits (upper and lower extremity motor, cognition, swallowing, speech, mood, spasticity, post-stroke neuropathic pain). Although the exact mechanism by which TMS improves post-stroke neurological deficits is inconclusive, the current status of TMS is at a critical period of translation of its value for clinical application. At this stage, it is necessary to document in detail the clinical efficacy, precautions, and adverse events of TMS for stroke, and to optimize and adjust the optimal stimulation parameters for TMS. However, TMS uses different stimulation parameters depending on the type of stroke, disease urgency, duration of disease, and post-stroke neurological sequelae, and perhaps even the timing of TMS intervention, which makes the optimal treatment protocol for various post-stroke sequelae difficult to find. Therefore, the optimal treatment protocol for TMS needs to be further explored with a large number and variety of stroke cases, a difficult process that requires small adjustments to various stimulation parameters and observation of efficacy. This process may be interrupted at any time due to patient non-cooperation, poor tolerance, financial constraints, and difficulties in follow-up, making it difficult to explore the optimal treatment protocol for stroke neurological deficits. Secondly, although there is a lot of basic research on TMS, the brains of experimental animals are relatively small compared to the human brain, and the TMS probe is about the same size as the probe used in the human brain in clinical practice, so can the TMS probe in animal studies be positioned as accurately as in the human brain? There is no evidence to support this yet. It is also one of the reasons why some researchers question the results of basic TMS studies. Therefore, the development of TMS probes that are more suitable for the head size of experimental animals is important to explore the potential molecular mechanisms of TMS treatment more precisely in the future.

## Author contributions

PZ and ZW developed the idea and revised the manuscript. LZ prepared and revised the manuscript. YJ, DW, YC, CZ, YP, NC, XY, SZ, RN, and PK reviewed the literature. All authors have read and approved the final manuscript.

## Funding

This study was supported by the National Natural Science Foundation of China (81960731 and 81860878), Yunnan Province biological medicine major special project (202102AA100016), Joint Special Project of Traditional Chinese Medicine in Science and Technology Department of Yunnan Province (2019FF002(-008), 202001AZ0700 01-002 and 030), Yunnan Province University Innovation Team Projects (2019YGC04), Yunnan province project Education Fund (2022Y372).

## Conflict of interest

The authors declare that the research was conducted in the absence of any commercial or financial relationships that could be construed as a potential conflict of interest.

## Publisher’s note

All claims expressed in this article are solely those of the authors and do not necessarily represent those of their affiliated organizations, or those of the publisher, the editors and the reviewers. Any product that may be evaluated in this article, or claim that may be made by its manufacturer, is not guaranteed or endorsed by the publisher.
